# Smart Operating Room in Digestive Surgery: A Narrative Review

**DOI:** 10.3390/healthcare12151530

**Published:** 2024-08-01

**Authors:** Vito Laterza, Francesco Marchegiani, Filippo Aisoni, Michele Ammendola, Carlo Alberto Schena, Luca Lavazza, Cinzia Ravaioli, Maria Clotilde Carra, Vittore Costa, Alberto De Franceschi, Belinda De Simone, Nicola de’Angelis

**Affiliations:** 1 Department of Digestive Surgical Oncology and Liver Transplantation, University Hospital of Besançon, 3 Boulevard Alexandre Fleming, 25000 Besancon, France; vlaterza@chu-besancon.fr; 2 Unit of Colorectal and Digestive Surgery, DIGEST Department, Beaujon University Hospital, AP-HP, University of Paris Cité, Clichy, 92110 Paris, France; 3 Unit of Emergency Surgery, Department of Surgery, Ferrara University Hospital, 44124 Ferrara, Italy; filippo.aisoni@ospfe.it; 4 Digestive Surgery Unit, Health of Science Department, University Hospital “R.Dulbecco”, 88100 Catanzaro, Italy; michele.ammendola@unicz.it; 5 Unit of Robotic and Minimally Invasive Surgery, Department of Surgery, Ferrara University Hospital, 44124 Ferrara, Italy; carloalberto.schena@ospfe.it (C.A.S.); nicola.deangelis@unife.it (N.d.); 6 Hospital Network Coordinator of Azienda Ospedaliero, Universitaria and Azienda USL di Ferrara, 44121 Ferrara, Italy; luca.lavazza@ausl.fe.it; 7 Azienda Ospedaliero, Universitaria di Ferrara, 44121 Ferrara, Italy; cinzia.ravaioli@ospfe.it; 8 Rothschild Hospital (AP-HP), 75012 Paris, France; mariaclotilde.carra@unife.it; 9 INSERM-Sorbonne Paris Cité, Epidemiology and Statistics Research Centre, 75004 Paris, France; 10 Unit of Orthopedics, Humanitas Hospital, 24125 Bergamo, Italy; vittore.costa@gavazzeni.it; 11 Department of Law, University of Ferrara, 44121 Ferrara, Italy; dfrlrt@unife.it; 12 Department of Emergency Surgery, Academic Hospital of Villeneuve St Georges, 91560 Villeneuve St. Georges, France; belinda.desimone@auslromagna.it; 13 Department of Translational Medicine, University of Ferrara, 44121 Ferrara, Italy

**Keywords:** operating room, surgery, general surgery, artificial intelligence, telesurgery, telemedicine, digestive surgery, hybrid operating room, augmented reality, robotic surgery

## Abstract

The introduction of new technologies in current digestive surgical practice is progressively reshaping the operating room, defining the fourth surgical revolution. The implementation of black boxes and control towers aims at streamlining workflow and reducing surgical error by early identification and analysis, while augmented reality and artificial intelligence augment surgeons’ perceptual and technical skills by superimposing three-dimensional models to real-time surgical images. Moreover, the operating room architecture is transitioning toward an integrated digital environment to improve efficiency and, ultimately, patients’ outcomes. This narrative review describes the most recent evidence regarding the role of these technologies in transforming the current digestive surgical practice, underlining their potential benefits and drawbacks in terms of efficiency and patients’ outcomes, as an attempt to foresee the digestive surgical practice of tomorrow.

## 1. Introduction 

The operating room (OR) represents one of the most essential resources of a hospital [1], accounting for more than 40% of hospitals’ total revenues. However, the OR is also the source of a large portion of hospitals’ expenses [1]. In the last decades, the volume and effectiveness of surgery have dramatically increased, with more than 300 million procedures performed in 2012 worldwide [2]. In this context, surgery-related adverse events, linked in a great proportion of cases to medical mistakes [3], represent the third leading cause of death in the United States [4]. 

The success of a surgical procedure requires high physical and cognitive efforts from the entire team of highly specialized professionals that are involved in the OR; the ability to anticipate decisions and consequences and the competence to act promptly are cardinal for the benefit of patients. Thus, improving OR efficiency and productivity is crucial to ensuring patients’ safety and cost reduction [5].

Recent surgical history was shaped by the introduction of new “technologies” leading to key transformations. First, the advent of general anesthesia extended the application of surgical procedures, and precision has been preferred over speed. Second, the introduction of the concepts of asepsis markedly decreased morbidity and mortality related to infections. Then, the introduction of minimally invasive surgery represented a third disruptor in surgical practice, being associated with faster recovery, shorter hospital stays, and overall better outcomes thanks to the implementation of video processors, low caliber instruments, new advanced energy sources, and master–slave robotic actuators in the OR [6]. We are now on the verge of the fourth paradigm shift, which is reshaping the OR of the future. The introduction of new technologies will allow streamlining workflows, enhancing communication and performance, and improving resource allocation, ultimately leading to better patient outcomes [7]. This surgical revolution will be characterized by smart assistance in physical, perceptual, and cognitive tasks in order to augment rather than replace surgeons [8,9,10]. 

Some key areas of surgical innovation are implemented from other high-stake, team-based sectors, such as aviation and car racing [11,12]: the introduction of OR black boxes [13] will allow performing postoperative analysis of errors and near misses, while the use of surgical control towers [14] will lead to real-time data analysis in order to coordinate operators on OR activities. The introduction of artificial intelligence and augmented reality into the operating theater is capable of augmenting surgeons’ cognitive capabilities thanks to computer vision and machine learning applications, as an attempt to mitigate human errors such as inadequate intraoperative decision-making or misidentification of anatomical variations [15]. Finally, other key innovations are the diffusion of hybrid OR [16], designed to facilitate real-time intraoperative imaging, and telesurgery [17], which leverages telecommunication technologies to extend surgical expertise beyond geographical boundaries. 

This narrative review aims to report the most recent evidence regarding the implementation of these smart technologies into current digestive surgical practice within the OR, and to provide insights on the transformative role that these innovations will play in shaping the OR and surgical practice of the near future. In particular, we will discuss the potential benefits and limitations of applying these technologies to enhance surgical efficacy.

## 2. Methods

Two authors (N.dA. and C.A.S.) queried the current literature through the PubMed (Medline) database on 10 May 2024 pertaining to the following topics: tower control; black box/surgical data recording; augmented reality; robotic operating room; hybrid operating room; telesurgery/telementoring. The queries and the number of articles found are reported in Supplementary Table S1. In order to synthesize a comprehensive review, the articles identified by this review were screened using the following inclusion criteria: published in PubMed-indexed journals; included information about the use of specific and available devices and/or technologies in digestive surgery. We included clinical trials, reviews, and case series regarding the optimization of surgical efficacy in the operating room thanks to new technologies represented by the main items of this review. After performing the search, two authors (V.L. and F.A.) identified pertinent articles by title and abstract screening. The cited references of each included publication were also screened in order to retrieve relevant information to the present study.

## 3. Surgical Control Tower and Black Box

Surgical errors leading to adverse events are correlated with high morbidity and mortality rates and occur mainly in the OR. Incidents play a crucial role in the overall OR time. A recent study demonstrated that the incidents observed inside the OR were associated with significant procedural delays, most of which are potentially preventable [18]. In addition, Inaba et al. [19] identified that for every additional 10 min of operative time in bariatric surgery operations, there were increased odds of 1-year mortality.

The studies by Nensi et al. [20] and Jung et al. [21] demonstrated that cognitive distractions (malfunctioning device, irrelevant conversation, surgical team member late or absent, management of other cases) and auditory distractions (door opening, machine alarm, loud noise, external communication) were extremely common in the OR, harming the surgeon’s precision and problem-solving ability, as well as increasing the likelihood of errors and adverse events. In addition, incidents that interrupt the OR workflow can alter the surgeon’s attention, consequently causing a worsening in the performance of a specific task. It is important to prevent errors in the surgical theater, as intraoperative mistakes may have serious consequences. Van Dalen et al. [22] explained that human error cannot be completely avoided, but the events that may lead to errors ought to be spotted early, analyzed, and reduced. This is possible through audio and video recording systems. These aim to assess communication, workflow, and education, beyond a wide range of surgical activities, like coaching moments between residents and surgeons or evaluating communication between surgeons and bedside assistants during robotic surgery [23,24]. In addition, there are sensor-based recordings to create teaching videos or to measure instrument kinematics [23]. These systems are connected to a control tower room, where it is also possible to examine the completion of surgery and to check the availability of the OR [25]. The control tower ensures real-time information on events and changes in the OR; this is important in order to modulate the workflow, subject to changes and unforeseen events, ensuring a smooth and efficient flow of patients [26].

There are many types of surgical data recording technology [27], which combine audio and video recording; currently, the most widespread in use is Black Box OR (ORBB) by Dr Teodor Grantcharov [28]. This elaborate system of cameras, microphones, and data recorders captures and synchronizes continuous sources of intraoperative data, providing the opportunity for retrospective case review, technical improvement, and error detection, with the aim of improving the quality of care. This system captures “technical skills”, such as intraoperative performance, and “non-technical skills”, including situation awareness, decision-making, teamwork, and leadership. ORBB was designed to be used to objectively record a procedure. This platform was able to observe a median of 20 errors and 8 events per case [21]. The OR checklist represents another proactive safety management system that can reduce intraoperative complications. It involves three stages: before the induction of anesthesia, before the surgical incision, and debriefing. The ORBB platform allowed assessment of checklist compliance in the OR and showed a significant reduction of complications when performed [29,30]. The postoperative multidisciplinary debriefing, instead, allows for the analysis of recorded surgical data and the identification of errors to prevent future occurrences [31].

In 2020 [32], the first description of an ORBB was published in the literature; it was used for the assessment of radiation protection in a hybrid angiography suite by combining objective measurements with behavioral analysis. Important deficiencies were demonstrated, such as the position of team members during angiography, the lack of communication, and the incorrect use of shielding equipment related to radiation protection. The authors underlined the importance of optimizing personal protective equipment and environmental shielding to minimize team radiation, such as maximizing the distance from the C-arm [33]. In Changhua County, Taiwan, an intelligent OR system has been developed that enables the transmission of surgical performance and intraoperative imaging data between the OR and the conference room [34], thus creating real-time communication, allowing interaction between the two environments and the possibility of exchanging views.

An important issue to be addressed, given the rapid expansion of the ORBB platform, is the regulation of the medico-legal aspect, the protection of health data, and the use of recorded surgical data. When the data are used for medical purposes for quality improvement or research, the information captured is managed by the hospital and physicians and would be protected from litigation, although in the case of a serious adverse event, resulting in a lawsuit, a judge might decide to ask the institution for the video data [27,35]. In addition, patient and caregiver privacy issues need to be taken into account when designing these systems, such as deidentifying the data so that they cannot be linked to the person. All of these issues affect the adoption of video technologies in hospitals. In this perspective, efforts are being made to create a transparent and comprehensive informed consent protocol for the patient so that the use of technology and how the data collected in these systems will be used can be defined.

## 4. Augmented Reality in the Operating Room

**Figure 1:**
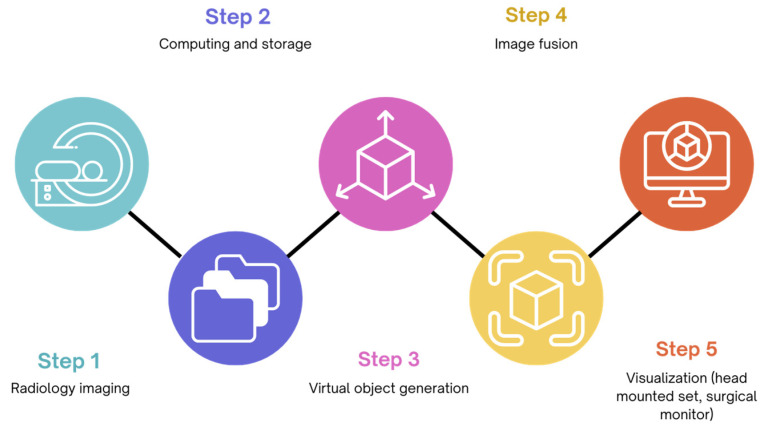
Flowchart representing the process of integrating AR during surgery to improve visualization.

Augmented Reality (AR) is a branch of Extended Reality (XR) that allows the integration of real-world and virtual-world information, by superimposing virtual content, mainly in the form of three-dimensional models, to real-time surgical images (Figure 1) [36]. The application of AR in the OR aims at strengthening human perception and complementing human information acquisition, in order to improve the safety and efficiency of the surgical procedure and avoid unintentional damage [37]. 

Since its conceptualization, the idea of an AR-aided surgery has been experimented in many surgical domains, such as neurosurgery and orthopedics [37], progressively making its way into clinical practice. 

More recently, AR has started to be applied also in abdominal surgery: pioneering research was published for its application during nephrectomy [38], pancreatoduodenectomy [39], and esophagectomy [40]. In recent years, it gained increasing recognition in the surgery of the parenchymal organs, particularly the liver, because of the complexity of its vasculature. Moreover, its application during minimally invasive surgery (MIS) may help overcome the intrinsic drawbacks of MIS, such as the lack or reduction of tactile feedback. AR started to permeate almost every domain of surgical practice: the creation of 3D models has been successfully employed in preoperative simulation for surgical planning [41] as well as in surgical skills training [42,43]. Additionally, AR has also been used in nursing, anesthesia, and patient education during the perioperative period [44,45].

However, real-time intraoperative navigation represents the real revolution of the surgery of the next future. In the last decade, several studies successfully tested AR’s efficacy in preoperatively planning liver resections strategy through 3D rendering, and then targeting the lesion and resection margins intraoperatively [46,47,48,49,50].

Buchs et al. [51], for example, managed to overlay the 3D preoperative images onto the live intraoperative stream and to add distance information to obtain a real-time guided transection. Then, after projecting the tumor image onto the liver surface, a visual indicator based on colors ranging from green to red alerted the surgeon to whether the resection was respecting or not the width of security margins defined before the operation. Moreover, the superimposition of pre-chemotherapy images has also been described as an aid in detecting vanishing metastasis, undetectable at preoperative or intraoperative images [52,53], reducing the time-wasting constant instrument shifting in case of difficulty. 

Another important application of AR is the identification of anatomical landmarks, such as arteries, veins, and biliary structures, in order to avoid intraoperative injuries, especially in the case of lesions located in critical areas such as the hepatic hilum [54].

In a study on 58 patients undergoing robotic cholecystectomy for gallbladder lithiasis, Diana et al. [55] identified 12 anatomical variants of biliary anatomy in eight patients, of which only seven were reported by preoperative radiology, thanks to the employment of AR. 

Another advantage of AR has been described for port placement in robotic liver surgery [56]. The authors projected an image of the liver on the patient’s skin surface using external landmarks, allowing the correct placement of the camera at the beginning of the operation and, after the pneumoperitoneum insufflation, of the other ports, with advantages in terms of improved manageability of the operator and total operative time duration. 

Zhang et al. [57] recently published their preliminary experience of the use of AR on 85 patients undergoing laparoscopic hepatectomies for primary liver cancer and found significantly lower intraoperative blood loss and blood transfusion rates during the resections where they used AR when compared to the conventional operation. Moreover, they also underlined an accelerated postoperative recovery and a significantly shorter length of hospital stay in the AR group. 

AR can be particularly useful to overcome the limitations of resecting lesions located in challenging areas, due to its ability to facilitate the identification of important anatomical structures. Zhu et al. [58] conducted a study on 76 patients to assess whether the combination of AR and indocyanine green (ICG) fluorescent imaging was beneficial for laparoscopic resection of centrally located hepatocellular carcinomas (HCCs). The authors found a significantly reduced intraoperative blood loss and transfusion frequency, shorter length of stay, and a lower rate of perioperative complication in patients undergoing hepatectomies with the combined use of AR and ICG when compared to those undergoing conventional laparoscopic hepatectomy. Additionally, they underlined a significantly reduced rate of conversion to laparotomy when AR and ICG were employed together (35.7% vs. 61.8%, *p* = 0.024).

In conclusion, the potentialities of AR in abdominal surgery are theoretically limitless and pave the way for a radical transformation of surgical procedures. Nevertheless, AR in abdominal surgery has shown a delayed development and distribution compared to other surgical fields, such as orthopedics, neurosurgery, and otolaryngology [59,60,61]. This may be due to anatomical characteristics, such as the deformation of soft organs according to the respiratory cycles and pneumoperitoneum creation [62]. In order to overcome these limitations, some strategies have already been described [63,64], and new platforms and software capable of shortening the time for model creation and improving the accuracy of overlapping images are making the development of AR in general surgery clinical practice more feasible [65]. Currently, despite the publication of more important series in the current literature, prospective comparisons are lacking. However, we can speculate that the application of AR will be enhanced by the widespread of robotic surgery, which represents an ideal platform to integrate advanced modalities of image-guided surgery in daily surgical practice.

## 5. Robotic Operating Room

The application of robotic surgery has been increasingly stable in the last decades in many surgical domains, such as neurosurgery, urology, and digestive surgery. Despite the improvements that robot-assisted surgery (RAS) has brought in terms of posture, visualization, and manipulation for the surgeon, it also has posed for the surgical team new challenges that may negatively influence the workflow of surgical practice [66]. Indeed, while the introduction of new surgical robotic platforms [67] has deeply modified the way of performing surgery, few changes have been made in the organization of the OR, which remained the same as for open and laparoscopic surgery. This brought technical and organizational issues in the OR. The increasing requirements of device management as well as personnel coordination raised the need for the adaptation of the OR devices as well as the OR personnel, in order to achieve a total integration of the whole OR tailored to robotic surgery requirements.

The introduction of new technologies has consequences not only for the operating surgeon but also for the whole team because of the changes in the division of labor [68]: communication, division of tasks, and especially the surgical environment need to adapt to the new conditions in order to limit the disruption of the operating room workflow, defined as the organization of activities that enables the provision of surgery [69]. Many flow disruptors impose a reorganization of the robotic operating room as imperative in order to overcome the new challenges that RAS introduced. 

Environmental factors, such as the spatial configuration of the OR, impose the team members to adjust to a different OR layout due to the presence of large robotic devices, potentially resulting in disorganization and obstruction [66]. A good workspace management with better configuration of the room layout can reduce the rate of collisions and obstructions as well as allow free movement. Many factors need to be taken into consideration in order to design an optimal layout of the OR: the characteristics and size of the patient cart, the surgical console, the vision cart, and all associated cables [70]. The cluttering of wires, tubes, and equipment may impair movement flow: Ahmad et al. found that half of all tracked operatory room movements are avoidable if the OR setup were optimized to accommodate team member tasks [71]. The authors underlined some critical areas of intervention to improve the room layout, including the optimization of monitor location, better access to the supply zone (that should also consider the sequence and frequency of equipment use), and facilitating unobstructed views. Moreover, the use of sliding doors, easier access to bins, the implementation of wireless transmission, and the development of an integrated operating table were other features that can improve OR efficiency. In order to address these needs, a model of robotic OR has been proposed, namely the Hyper SCOT suite [72]. The Hyper SCOT OR was first introduced in 2019 and was intended for neurosurgery. It is equipped with cutting-edge technology, such as robotic technology and AI compatibility, and new approaches in terms of architectural design. This OR features an optimal layout: monitors are placed according to a layout that allows the surgeon facing the operative field to obtain all desired information at a glance, and in a comfortable way for medical staff to check intraoperative information. Flat and frameless wall panels are designed for optimal focus on the treatment, and control devices and pipelines are hidden behind doors. A modulable OLED lighting system is capable of adapting to any need (for example, warm lighting for reducing patients’ anxiety, high illumination for cleaning, and efficient preparation for surgery). A robotic operating table was also implemented, in order to improve the efficiency of patient transfer in case of intraoperative imaging. Finally, a communication interface can show intraoperative information, device data, in-room camera images, and intraoperative images at the same time.

Besides integrated robotic ORs, new types of robotic “assistants” other than surgical robots have been introduced to enhance the quality of surgery and efficiency in the OR. A robotic armrest, called iArmS [73], was developed in 2015 to assist the surgeon’s arm during precise tasks and to reduce fatigue. It is totally passive with no actuator, and it can freely move with the surgeon’s arm or help the operator maintain the position thanks to electric brakes activated by feedback from force sensors. It was tested by 14 neurosurgeons [73] with an 86% decrease in tremors. As regards lighting, intelligent lighting systems capable of targeting a region of interest and being modulated in terms of intensity and colors by hand or vocal gestures are already a reality [74,75]. As regards robotic lighting systems [76], featuring the same function but being mounted on a robotic arm providing more options in terms of lighting position and direction, their feasibility has already been discussed but they are not currently employed in clinical practice.

## 6. Hybrid Operating Room

Hybrid operating rooms (HORs) are surgical theaters equipped with radiological imaging systems, such as fluoroscopy, computed tomography (CT), or magnetic resonance imaging (MRI) devices [77], which enable the integration of surgical and interventional radiological procedures in a single setting. The first clinical employment of HORs was reported in cardiothoracic surgery [78], being then gradually implemented in other surgical endeavors such as neurosurgery, vascular surgery, and orthopedic surgery [16,79].

In conventional ORs, surgeons usually rely on preoperative imaging to identify anatomical structures and lesions. Conversely, intraoperative imaging is able to provide a real-time depiction of reality, helping to improve surgical precision and therefore ameliorating patients’ outcomes [80,81]. Several studies have already demonstrated that HOR employment increases safety and efficiency [82,83]. Imaging technologies may be particularly beneficial during minimally invasive procedures, whereby visualization, access, and haptic feedback are often limited.

As regards general and visceral surgery, hepatobiliary and trauma surgery represent the two fields in which HORs have been experimented and may find interesting applications in daily clinical practice.

Regarding liver surgery, HORs may provide the possibility of combining the surgical resection with other minimally invasive radiological procedures. An interesting application of this concept is as an attempt to shorten the interval between resections during a two-stage hepatectomy, as proposed by Odisio et al. [84]. In the so-called fast track two-stage hepatectomy, the authors proposed to perform the portal vein embolization during the first resection in a HOR, demonstrating its feasibility and safety in a cohort of 19 patients [85]. They found no complications deriving from the combination of the two procedures, showing at the same time a shortened time lapse between the two resections and to adjuvant chemotherapy compared to the intervals reported in the literature [86,87].

The usefulness of HORs has also been demonstrated in a case of hemorrhagic traumatic liver laceration by Hagiwara and colleagues [88]: during the same operation, the surgeons were able to perform the perihepatic packing and the Pringle maneuver, while at the same time the interventional radiologist was able to perform the transarterial embolization, sparing precious time in the treatment of a patient in critical conditions [89]. 

Other authors have tested the utility of HORs in guiding the surgeon by the identification of the bleeding vessel in traumatic liver injury [90]; addressing portal vein complications following liver transplantation [91]; helping the surgeon solve problems related to hepatic lesions with vascular involvement, such as monitoring the effect of a cyst fenestration on the degree of vascular decompression [92]; and facilitating the resection of a giant hemangioma by intraoperative embolization [93]. 

The availability of HORs may also provide additional imaging modalities in order to facilitate intraoperative orientation during minimally invasive hepatic resections. Ueno et al. [94] used HOR to guide the selective pedicular injection of ICG in 10 patients in order to precisely guide the resection of anatomical segments. Other teams tested the fusion of three-dimensional models derived from preoperative CT scans with intraoperative images in order to improve the safety and the oncological outcomes of liver resections: Kenngott et al. [63] used intraoperative cone beam CT imaging as a navigation tool during liver surgery. They combined three-dimensional models and intraoperative fluoroscopic images to localize the tumor in relation to the surgical instruments, with a 2.5 mm maximum error. The use of intraoperative fluoroscopy was successfully replicated in other series [95,96].

Nevertheless, real-time integration of liver models with surgical images has proven challenging due to the constant deformation of tissues due to manipulation and movements. Many authors [97,98,99,100,101] suggested several methods to realign the optical view with the three-dimensional liver models using intraoperative CT imaging, improving in this way the accuracy of augmented reality (AR) imaging in animal models, but only in an experimental setting so far.

Finally, while no studies have been published reporting combined resection and ablation of liver lesions using intraoperative CT or MRI imaging, some pioneering studies [102,103,104,105,106,107] established its potential feasibility in order to economize in terms of anesthesia time and length of hospital stay compared to two separate procedures.

As regards HOR in trauma surgery, its application could bring significant advantages especially in abdominopelvic injuries, where various bleeding control techniques are required, such as laparotomy, angiographic embolization, and orthopedic fixation. The employment of a Trauma Hybrid Operating Room (THOR) may allow rapid bleeding control and reduce patient transportation time. A recent study [108] analyzed the procedure time and mortality of 56 abdominopelvic trauma patients who required both surgery and interventional radiology in separate environments compared to 35 patients who received the same treatments in THORs. The authors found that the THOR group underwent significantly less thoracotomy, more resuscitative endovascular balloon occlusion of the aorta (REBOA), and more pelvic angiographic embolization. Moreover, the THOR group showed a significantly shorter procedure time, which was similar excluding the transit time in the conventional treatment group. While mortality was similar in both groups, the mortality deriving from exsanguination was significantly lower in the THOR group. 

Overall, the widespread of HORs started to transform current surgical practice not only by implementing novel treatment modalities but also by improving the safety and efficacy of existing treatments. The availability of intraoperative imaging eliminates the need for patient transfers and makes possible real-time assessment of operative results. Merging radiological and surgical environments may significantly reduce anesthesia time and total procedure length [80,109,110]. Nevertheless, the use of HORs has some economic, safety, and logistical concerns.

First, a HOR is considerably more costly than a conventional operating room. Patel et al. calculated a EUR 19.88 cost per minute for the HOR compared to EUR 9.45 for conventional OR [111]. However, combining two or more interventions can save time in terms of patient preparation, positioning, anesthesia induction, and recovery. Second, the employment of intraoperative imaging may increase the exposure to ionizing radiation for both the patient and operating room personnel. Moreover, the use of precautions such as protective clothing and radiation protection shields may be inconvenient in the context of a surgical operation. Third, the integration of different procedures may bring logistical challenges and intraoperative workflow disruptions, requiring specific training for the whole multidisciplinary team [112,113].

## 7. Telesurgery and Telementoring 

Telemedicine is defined as a subset of e-health that utilizes communication networks to facilitate the delivery of services and medical expertise between remote sites [114]. While successfully applied for surgical consultations during the COVID-19 pandemic period due to infection control restrictions [115], telemedicine may have its most interesting application in the form of telesurgery and telementoring.

The concept of telesurgery, or remote surgery, meaning the existence of physical distance between the surgeon and the patient, had already been explored in the 1970s by the U.S. National Aeronautics and Space Administration (NASA), which was interested in treating astronauts in outer space [116]. Simultaneously, the advent of robotic surgery and the significant advancements in telecommunications led to the first telerobotic surgery, called the “Lindbergh Operation”, successfully performed on 7 September 2001, by Professor Jacques Marescaux on a patient in Strasbourg, France.

It was a telesurgery-assisted cholecystectomy, completed in 54 min without complications [117]. Despite the Lindbergh Operation being a symbolic milestone in surgery, the development of remote surgery was not possible in clinical practice until the integration of new technologies, such as the fifth-generation (5G) internet, that allowed the significant limitations of the network system to be overcome [118]. During the Lindbergh Operation, Marescaux and colleagues worked with a resolution of 1024 by 768 pixels and a data transfer rate of 10 Mbps, allowing a maximum latency of 330 ms. Since then, the improvement in terms of video quality and data transfer speed has led to several other successful telesurgeries [119].

Nowadays, the DaVinci robot is capable of at least 1920 by 1080 pixel resolution, and 5G networks can both reduce the latency, defined as the response delay between a device and the hosting server or target device, and improve video stream quality up to 10 Gb/s. Recent data recorded during telesurgery operations reported, at latencies ranging between 76 and 150 ms, a response time of 475–660 ms for surgeons [120,121], which is comparable to the response time found by Zheng et al. in 2003 during “traditional” endoscopic surgery (397 ± 19 ms) [122].

The implementation of telesurgery in everyday clinical practice may make the collaborations between different surgical experts from various healthcare centers possible in real time: a patient could benefit from the expertise of more than one specialist simultaneously, with great advantages in terms of quality of care and time optimization [123,124]. In addition, the widespread incorporation of these new technologies could fill the gap in terms of surgical expertise between metropolitan and rural locations, thanks to the planned 5G covering of 90% of rural America in the next few years as revealed by a report published by T-Mobile in *Forbes* [125]. Some studies analyzing prostate [126] and bladder [127] cancer patients requiring robotic surgical procedures have shown a treatment delay due to increased patient travel distance, with odds of traveling a medium to long range of 1.1–1.2 times higher odds in prostate cancer patients requiring robot-assisted radical prostatectomy compared to open. Finally, on a greater scale, they could lead to fulfilling the need for surgical expertise in developing countries worldwide [128,129,130].

Technological advances can also respond to the unmet need for adequate mentoring in minimally invasive surgery, specifically in the form of telementoring. Telementoring is defined as the possibility of an expert surgeon actively observing and supervising in real time a procedure performed by another surgeon in a different institution. 

This is particularly important to mitigate the adverse clinical and economic effects that the implementation of new technologies, such as robotic surgery, could have on patients’ outcomes. Moreover, the learning curve for minimally invasive surgery is estimated to be longer than that of open surgery [131]. On this basis, it is of primary importance to optimize patients’ outcomes during the learning curve by adopting rigorous mentoring programs for trainees. Insufficient training in robotic surgery was identified as one of the top 10 risks to patients in a review by the Emergency Care Research Institute (ECRI) about the dangers of health technologies [132].

In particular, telementoring can be classified into four different levels according to the increasing level of interaction between the mentor and the trainee: verbal guidance, when the mentor guidance is limited to verbal instructions while watching a real-time video of the operation; telestration, when the tutor can guide the trainee indicating target areas on the screen; teleassistance, when the mentor can assume the role of an assistant by taking control of one or more robotic surgical instruments; and, finally, telesurgery, when the tutor has the freedom of actually performing the whole operation remotely [133].

Interesting data suggesting a safety profile and effectiveness for telementoring similar to traditional tutoring come from a recent systematic review including 66 studies, of which 22 (33%), 24 (36%), and 20 (30%) reported the implementation of telementoring within the same hospital, outside the institution and outside the country, respectively. Of twelve studies that directly compared remote tutoring with traditional face-to-face tutoring, seven showed no differences between remote tutoring and in-person tutoring, and no study found worse postoperative outcomes for telementoring [133].

On this basis, telementoring may represent a practical and economically profitable option to promote the teaching of minimally invasive surgical techniques around the world, in order to overcome the limited access to specific fellowship programs and to provide high-level surgical care in medically underserved regions.

## 8. Recommendations for Future Research: Is Artificial Intelligence (AI) the Answer?

As emerged in the previous sections, recent advancements are transforming the operating room from an analog space into a highly digitized and integrated environment. At present, these innovations are mostly used in an experimental phase. They tend to lack flexibility and to be task-specific, with few demonstrated interconnected implementations. 

For these reasons, the primary aim of future research should be how to integrate these technologies into a cohesive ecosystem [134].

In order to accomplish this goal, one key technology involved could be generative artificial intelligence (GAI). Medical GAI models might be able to collect data from a variety of inputs such as imaging, any spoken or written information from medical staff, analytics, or any signal detected from device feedback or wearable devices and combine them with specific medical knowledge from the current literature. They might then be capable of generating a highly diverse output, including augmented procedures, automated reports generation, and chatbots for verbal communication [135]. This versatility in terms of input and output could represent the foundation for the evolution of smart operating theaters.

Large language models (LLMs) will potentially be the bridge between AI and surgery, representing the communication modality that makes AI-assisted surgery real. For example, the integration of LLMs into surgical robots and AR systems could be able to provide real-time guidance during surgical operations. Andras et al. [136] have already demonstrated the capabilities of tension sensors and AR in enhancing the surgical experience: the addition of LLMs could allow for real-time communication able to provide feedback or even step-by-step instructions about a specific surgical procedure. This application would overcome the simple use in simulation or training, limited by the absence of adaptive feedback and the low case variability [137].

Additionally, LLMs open up entirely new possibilities for providing feedback and enhancing surgical training. In recent years, AR has been employed to deliver novel tools for surgical performance metrics assessment, especially during MIS [138]. Therefore, data from multiple modalities, such as functional near-infrared spectroscopy, video console input, eye tracking, and kinematic data, could be used as input to generate scores on the base of different machine learning methods [139]. LLMs can potentially enhance the accessibility and comprehensibility of these scores, providing feedback that can be delivered not only after the surgical procedure but also in real-time during surgical interventions. Moreover, feedback provided by LLMs can be adaptive, highlighting gaps in the data or accuracy issues [140]. The responses could be instantly recorded by the LLM, enabling progress tracking and revisions with mentors. Real-time oral feedback from surgeons could also be captured and stored for later review [141]. Future developments may use this feedback for educational purposes, by creating a global repository of evaluations from surgeons worldwide that are accessible to surgical trainees worldwide. Ultimately, this could improve surgical performance and patients’ outcomes, and consequently reduce costs.

Another groundbreaking field of future research is autonomous robotic surgery, meaning the capability of robotic systems to perform surgical operations with a high degree of autonomy [142]. While still in an experimental context, preliminary results have shown the potential to improve surgical outcomes thanks to the integration of AI and robotics. The Smart Tissue Autonomous Robot (STAR) matched and in some cases outperformed surgeons in ex vivo intestinal anastomosis. STAR is an autonomous robot capable of performing the procedure after human approval. It was initially tested in 2016 on phantom bowels, making fewer mistakes and showing more efficacy compared to human surgeons [143]. In the scale of autonomy that surgical robots may achieve [144], STAR is considered a level 3 out of 5 (meaning a robot capable of understanding a surgical scenario, planning, and executing a specific task). Currently, no system has yet reached level 5, meaning the capability of performing surgery without human assistance. Whenever this becomes a reality, regulations will become mandatory to provide safety, confidence, and transparency in these novel systems and to solve important ethical questions in terms of accountability, liability, and culpability [145].

## 9. Conclusions with Ethical and Legal Aspects

**Figure 2:**
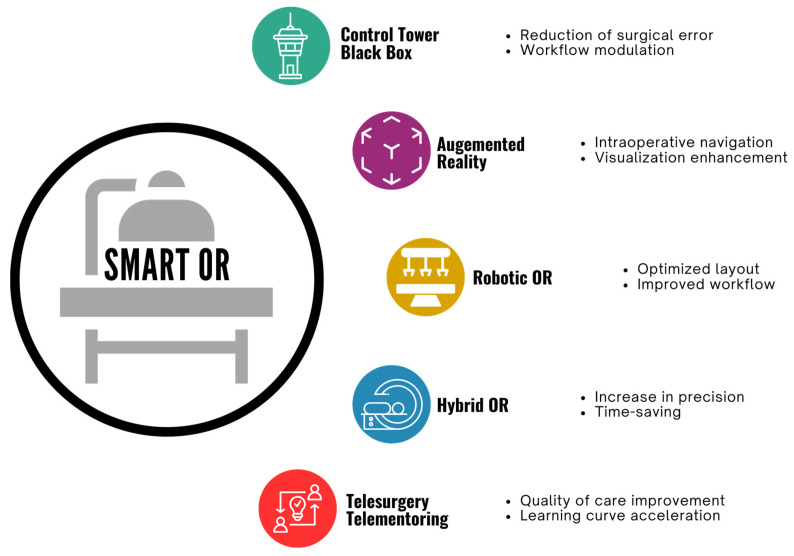
Schematic representation of the transformative role of smart technologies in reshaping the OR and surgical practice of the near future.

Historically, the field of surgery has been relatively cautious with the employment of potentially disruptive technologies [146]. Nevertheless, the potential for new digital technologies to improve surgical care suggests that their incorporation into daily practice is inevitable. As a result, improved data acquisition, fast telecommunications, computer vision, and augmented reality have the potential to reshape surgery and the surgical OR in the near future (Figure 2). However, these great opportunities also carry some drawbacks, and specific considerations are needed before their implementation. 

The first important concern is related to privacy and information security. The growing adoption of connected technologies and devices in the OR, as well as the recording of intraoperative data to develop machine learning (ML) models, requires a large amount of surgeon biometrics, surgical videos, and instrument kinematics, often shared across multiple institutions. This makes hospitals susceptible to targeted attacks that can cause patient harm due to the leak of sensitive data. Some reports have also shown that the number of cyberattacks on hospitals has been significantly increasing [147]. For this purpose, Gordon et al. [148] explained how health systems can limit the effects of cybersecurity attacks in OR by minimizing real-time connectivity during procedures, establishing downtime procedures, and collaborating with enterprises on developing safety measures, as well as using standardized ways to anonymize data from collection to usage [149]. 

Concerning telesurgery, the idea of getting medical care from a surgeon who has no real interaction with the patient can generate a degree of skepticism as well as legal and ethical issues, as laws differ across the state and country borders [150]. Moreover, as different centers collaborate during telesurgery, some concerns may also regard the distribution of billing and responsibility to the participating hospitals [123].

Additionally, the employment of AR technology introduces new challenges that need to be addressed. Registration and tracking errors, as well as obscured operating fields or other malfunctions of the technology, could impact surgeons’ ability to perform correctly. This raises questions about the consequences of relying on such technology, and the need to preserve surgical proficiency and competence without overreliance on nonhuman aids. This also raises accountability problems: it is not clear who would bear responsibility in case of negative outcomes due to technology failure, as it should be distributed among various parties such as the surgeon, the hardware and software provider, and the designer of the algorithms [151].

Finally, the impact of these new tools may extend beyond the operating theater. As regards the assessment of surgeons’ performance by AI models, while it may enhance the comparability of surgeons’ skills for both insurance providers and patients [138], empowering patients to select surgeons according to their own benefit, further technical advances are needed to reduce under- and over-skilling bias and to make these models more transparent before enabling global surgeon credentialing [152]. Additionally, as surgery outcomes depend on a variety of factors, such as the expertise of the complete operating team, patient factors, and the level of perioperative care, filtering the contribution of the surgeon to a specific surgical procedure remains challenging [153]. Currently, AI models often fail in terms of accuracy, and their adoption in surgical practice may still be limited by the absence of domain-specific LLMs [154]. 

## Data Availability

Not applicable.

## Supplementary Materials

The following supporting information can be downloaded at: https://www.mdpi.com/article/10.3390/healthcare12151530/s1. Supplementary Table S1. Research queries for each included item and the number (N) of articles found.
