# Supplementary material for: Smart Operating Room in Digestive Surgery: A Narrative Review
Source: Healthcare (Basel). 2024 Aug 1;12(15):1530. doi: 10.3390/healthcare12151530 (PMC11311806; doi:10.3390/healthcare12151530)
Supplement: Supplementary file 1 [file healthcare-12-01530-s001.zip › healthcare-3108516-supplementary.pdf]

## Supplementary material

| Supplementary Table 1. Research queries for each included item and the number (N) of articles found | | |
|---|---|---|
| **Item** | **Query** | **N** |
| **Tower control** | ((tower control[Title/Abstract]) OR (control room[Title/Abstract])) AND ((((((((operating room[MeSH Terms]) OR (operating theater [MeSH Terms])) OR (operating room[Title/Abstract])) OR (operating theater[Title/Abstract])) OR (surgery[Title/Abstract])) OR (surgical procedure[Title/Abstract])) OR (surgical intervention[Title/Abstract])) OR (operating room[Title/Abstract])) | 21 |
| **Black box/surgical data recording** | ((((((((operating room[MeSH Terms]) OR (operating theater [MeSH Terms])) OR (operating room[Title/Abstract])) OR (operating theater[Title/Abstract])) OR (surgery[Title/Abstract])) OR (surgical procedure[Title/Abstract])) OR (surgical intervention[Title/Abstract])) OR (operating room[Title/Abstract])) AND ((((((black box[Title/Abstract]) OR (black box technology[MeSH Terms])) OR (black box technology[Title/Abstract])) OR (surgical data recording[Title/Abstract]))) | 148 |
| **Augmented reality/virtual reality** | ((((((((operating room[MeSH Terms]) OR (operating theater [MeSH Terms])) OR (operating room[Title/Abstract])) OR (operating theater[Title/Abstract])) OR (surgery[Title/Abstract])) OR (surgical procedure[Title/Abstract])) OR (surgical intervention[Title/Abstract])) OR (operating room[Title/Abstract])) AND ((((augmented reality[Title/Abstract]) OR (virtual reality[Title/Abstract])) OR (augmented reality[MeSH Terms])) OR (virtual reality[MeSH Terms])) Filters: from 2014 - 2024 | 2945 |
| **Robotic operating room** | (Robotic[Title/Abstract]) AND (operating room[Title/Abstract]) | 1070 |
| **Hybrid operating room** | (Hybrid[Title/Abstract]) AND (operating room[Title/Abstract]) | 629 |
| **Telesurgery/telementoring** | (((((telesurgery[Title/Abstract]) OR (telementoring[Title/Abstract])) OR (telesurgery[MeSH Terms])) OR (remote surgery[MeSH Terms])) OR (remote sugery[Title/Abstract])) AND ((((((((operating room[MeSH Terms]) OR (operating theater [MeSH Terms])) OR (operating room[Title/Abstract])) OR (operating theater[Title/Abstract])) OR (surgery[Title/Abstract])) OR (surgical procedure[Title/Abstract])) OR (surgical intervention[Title/Abstract])) OR (operating room[Title/Abstract])) Filters: from 2014 - 2024 | 1669 |
